# Disorganized attachment in adolescence: Emotional and physiological dysregulation during the Friends and Family Interview and a conflict interaction – Erratum

**DOI:** 10.1017/S0954579421000109

**Published:** 2021-08

**Authors:** Alessandro Decarli, Blaise Pierrehumbert, André Schulz, Violetta Katharina Schaan, Claus Vögele

As a result of a production error, in Decarli et al. 2020, figures 3 and 4, referenced in the text as online supplementary material, in fact also appear in the main article.

The publisher regrets this error.
